# The Association Between Metabolically Healthy Obesity and the Risk of Proteinuria: The Kansai Healthcare Study

**DOI:** 10.2188/jea.JE20170082

**Published:** 2018-08-05

**Authors:** Shinichiro Uehara, Kyoko Kogawa Sato, Hideo Koh, Mikiko Shibata, Shigeki Kinuhata, Akiko Yamada, Keiko Oue, Hiroshi Kambe, Michio Morimoto, Tomoshige Hayashi

**Affiliations:** 1 Preventive Medicine and Environmental Health, Osaka City University Graduate School of Medicine, Osaka, Japan; 2 Hematology, Osaka City University Graduate School of Medicine, Osaka, Japan; 3 Medical Education and General Practice, Osaka City University Graduate School of Medicine, Osaka, Japan; 4 Osaka City University Hospital, Osaka, Japan; 5 Kansai Health Administration Center, Nippon Telegraph and Telephone West Corporation, Osaka, Japan

**Keywords:** metabolically healthy obese phenotype, metabolically healthy obesity, proteinuria, chronic kidney disease, prospective study

## Abstract

**Background:**

Metabolically healthy obesity seems to be a unique phenotype for the risk of cardiometabolic diseases. However, it is not known whether this phenotype is associated with the risk of proteinuria.

**Methods:**

Study subjects were 9,185 non-diabetic Japanese male workers aged 40–55 years who had no proteinuria, an estimated glomerular filtration rate ≥60 mL/min/1.73 m^2^, no history of cancer, and no use of antihypertensive or lipid-lowering medications at baseline. Obesity was defined as body mass index ≥25.0 kg/m^2^. Metabolic health was defined as the presence of no Adult Treatment Panel III components of the metabolic syndrome criteria, excluding waist circumference, and metabolic unhealth was defined as the presence of one or more metabolic syndrome components, excluding waist circumference. “Consecutive proteinuria” was considered positive if proteinuria was detected twice consecutively as 1+ or higher on urine dipstick at annual examinations to exclude chance proteinuria as much as possible.

**Results:**

During the 81,660 person-years follow-up period, we confirmed 390 cases of consecutive proteinuria. Compared with metabolically healthy non-obesity, metabolically healthy obesity was not associated with the risk of consecutive proteinuria (multiple-adjusted hazard ratio [HR] 0.86; 95% confidence interval [CI], 0.37–1.99), but metabolically unhealthy non-obesity with ≥2 metabolic syndrome components (HR 1.77; 95% CI, 1.30–2.42), metabolically unhealthy obesity with one component (HR 1.71; 95% CI, 1.12–2.61), and metabolically unhealthy obesity with ≥2 metabolic syndrome components (HR 2.77; 95% CI, 2.01–3.82) were associated with an increased risk of consecutive proteinuria.

**Conclusions:**

Metabolically healthy obesity did not increase the risk of consecutive proteinuria in Japanese middle-aged men.

## INTRODUCTION

Chronic kidney disease (CKD) is one of the major public health problems all over the world. As proteinuria, which is one criteria of the definition of CKD, is a risk factor of cardiovascular diseases^1^^,^^2^ and end-stage renal disease,^3^^,^^4^ it is important to identify the risk factor of proteinuria to delay or prevent the incidence of proteinuria.

Although obesity is a known-risk factor for not only proteinuria but also cardiometabolic diseases, all obese subjects do not develop these diseases. One of the obese phenotypes, metabolically healthy obesity, has been focused on in association with the risk of cardiometabolic diseases.^5^^–^^7^ However, it is not known whether this phenotype is associated with the risk of proteinuria. Only one retrospective cohort study, but no prospective cohort study, was available on relating metabolically healthy obesity with the risk of proteinuria.^8^ As authors defined metabolic health as metabolic syndrome components ≤1, all subjects classified as having metabolically healthy obesity were not really metabolically healthy.

Previous epidemiological studies regarding metabolically healthy obesity have raised an important issue: there is no accepted criteria for metabolically healthy obesity.^9^^–^^11^ Some studies have defined metabolic health as metabolic syndrome components ≤1, while others have defined metabolic health as metabolic syndrome components ≤2. Therefore, to accurately evaluate the association between metabolically healthy obesity and the risk of proteinuria, metabolic health should be defined as no metabolic syndrome components other than waist circumference and metabolic unhealth should be defined as metabolic syndrome components other than waist circumference ≥1. The purpose of this study was to prospectively examine the association between metabolically healthy obesity and the risk of proteinuria during the 11-year observation period.

## MATERIAL AND METHODS

### Study population

The Kansai Healthcare Study is an ongoing cohort investigation that has been designed to clarify the risk factors for cardiometabolic diseases.^12^^,^^13^ Between April 2000 and March 2001, 12,647 male workers of a company in the Kansai region of Japan were enrolled in this study. The subjects were aged 40–55 years and had sedentary work at entry. All employees of this company aged 40 years or older undergo detailed annual medical check-ups. Japanese law requires all employers to conduct annual health screenings for all employees. When data of a cohort study are based on these annual health screenings, Japanese ethical guidelines for epidemiological research state that informed consent from participants does not need to be obtained. This study was approved by the Human Subjects Review Committee at Osaka City University.

Of the 12,647 in the original cohort, we excluded 1,956 subjects who had proteinuria, an estimated glomerular filtration rate (eGFR) <60 mL/min/1.73 m^2^, and who had a fasting plasma glucose ≥126 mg/dL or were taking hypoglycemic medications or insulin. We also excluded 841 men who were taking antihypertensive medications or oral lipid-lowering medications, or who had cancer at entry. We also excluded 312 men who did not undergo medical check-ups during the follow up period and 353 men because of missing variables. Thus, the analyzed final study sample consisted of 9,185 men.

### Data collection and measurements

We collected the data at the annual clinical examination. The data consisted of medical history, physical examination, anthropometric measurements, self-reported questionnaires about lifestyle characteristics, measurements of blood samples, and dipstick urinalysis. Trained nurses carried out all measurements. Urine samples were collected as clean-catch, mid-stream, and random urine specimens. The results of the dipstick urinalysis were interpreted as negative, ±, 1+, 2+, 3+, or 4+. Results of ± or negative on dipstick urinalysis were defined as normal. Blood samples were collected after a 12-hour overnight fast. Triglycerides, high-density lipoprotein (HDL) cholesterol, fasting plasma glucose, and serum creatinine were measured with fasting blood samples. Triglycerides, HDL-cholesterol, and serum creatinine were measured using a Hitachi 7350 automatic chemistry analyzer (Hitachi Ltd., Tokyo, Japan); an enzymatic method for serum creatinine, an enzymatic method for triglycerides, and a direct assay were used for HDL-cholesterol. Serum creatinine was measured using the Jaffe method in 1,799 subjects at the baseline examination; the Jaffe values were recalibrated to correspond to enzymatic values using the following equation developed by the analytical laboratory: serum creatinine (mg/dL, enzymatic method) = 1.02 × serum creatinine (mg/dL, Jaffe method) − 0.25 (*r* = 0.9996). Then we calculated eGFR using the Modification of Diet in Renal Disease equation for Japanese persons, which has been validated by the standard inulin clearance technique, as follows: eGFR = 194 × age^−0.287^ × serum creatinine^−1.094^ (mg/dL, the enzymatic method).^14^ Systolic blood pressure (SBP) and diastolic blood pressure (DBP) were measured in a sitting position with an automated sphygmomanometer (BP-203RV; Omron Colin, Tokyo, Japan, and Udex-super; ELK, Osaka, Japan) after approximately 5 minutes or more of rest. The Spearman’s correlation between the former device and a standard mercury sphygmomanometer was 0.985 for SBP and 0.976 for DBP. The Spearman’s correlation between the latter device and a standard mercury-column sphygmomanometer was 0.997 for SBP and 0.976 for DBP. Hypertension at baseline was defined as systolic blood pressure ≥140 mm Hg or diastolic blood pressure ≥90 mm Hg.^15^ Body mass index (BMI) was calculated as the weight in kilograms divided by the square of the height in meters. The self-reported questionnaires about lifestyle characteristics consisted of questions regarding smoking habits, drinking habits, and regular leisure-time physical activity. For smoking habits, subjects were categorized as non-smokers, past smokers, or current smokers. The questions about drinking habits included the number of drinking times per week and the usual amount of alcohol consumed each time according to Japanese standard drinks. Average alcohol consumption was calculated as (the quantity consumed per drinking day) × (the weekly frequency of drinking)/7. Average daily alcohol consumption was classified into four groups: non-drinkers, 0.1–23.0, 23.1–46.0, and ≥46.1 g ethanol/day. One Japanese standard drink is 23 g of ethanol. To assess leisure-time physical activity, a single question was used with three possible answers: rarely, sometimes, or regularly (at least once a week). Subjects were classified as engaging in leisure-time physical activity at least once weekly or less than once weekly. The validity of this questionnaire has been described in detail previously.^12^

Metabolic health was defined as having no metabolic syndrome components, as defined by the Adult Treatment Panel III, excluding waist circumference.^16^ Metabolic unhealth was defined as having at least one component form the Adult Treatment Panel III other than waist circumference. The components of the Adult Treatment Panel III metabolic syndrome criteria other than waist circumference were: 1) triglycerides ≥150 mg/dL, 2) HDL-cholesterol <40 mg/dL, 3) SBP ≥130 mm Hg and/or DBP ≥85 mm Hg, or 4) fasting plasma glucose ≥100 mg/dL.^17^ Obesity was defined as BMI ≥25.0 kg/m^2^ for the Asian criteria of obesity.^18^ Therefore, six phenotypes using metabolic health and obesity status were defined: 1) metabolically healthy non-obesity: no metabolic syndrome component with non-obesity; 2) metabolically unhealthy non-obesity (metabolic syndrome component =1): one metabolic syndrome component with non-obesity; 3) metabolically unhealthy non-obesity (metabolic syndrome components ≥2): two or more metabolic syndrome components with non-obesity; 4) metabolically healthy obesity: no metabolic syndrome component with obesity; 5) metabolically unhealthy obesity (metabolic syndrome component =1): one metabolic syndrome component with obesity; 6) metabolically unhealthy obesity (metabolic syndrome components ≥2): two or more metabolic syndrome components with obesity.

### Definitions of outcome

Proteinuria was defined as 1+ or higher (30 mg/dL or higher)^19^ on urine dipstick urinalysis at the annual medical check-up. In this study, we used two definitions of proteinuria. One was consecutive proteinuria, which was defined as proteinuria detected twice consecutively during the follow-up period to exclude transient proteinuria as much as possible. The other was persistent proteinuria, which was defined as proteinuria detected at every annual medical check-up since the first detected proteinuria.

### Statistical analysis

Baseline characteristics were expressed as the mean (standard deviation), median (interquartile range), or frequencies (%). Differences in baseline characteristics between subjects who developed consecutive proteinuria and those who did not develop it were determined using a *t*-test for normally distributed continuous variables, Mann-Whitney test for non-normally distributed continuous variables, and a chi-squared test for categorical variables. Multivariate Cox proportional hazards models were used to investigate the risk of developing consecutive and persistent proteinuria in relation to the six phenotypes of the metabolically healthy and obese status. Follow-up of the subjects was continued until an outcome was detected or until the 11-year follow-up examination (between April 1, 2011 and March 31, 2012), whichever came first. In all multivariate models, nonlinear effects of continuous independent variables were also evaluated using quadratic, square root, and log transformations, which were tested in the multivariate Cox proportional-hazards model to determine if these improved the fit of the linear models. Of all continuous independent variables in all the multivariate models, average daily alcohol consumption did not fit the linearity assumption; therefore, we fitted models using average daily alcohol consumption as a categorical variable: non-drinkers, 0.1–23.0, 23.1–46.0, and ≥46.1 g ethanol/day. Multivariate models were adjusted for age, eGFR, smoking habits (non-smoker, past smoker, or current smoker), regular leisure-time physical activity (yes/no), and average daily alcohol consumption (non-drinkers, 0.1–23.0, 23.1–46.0, and ≥46.1 g ethanol/day) at baseline. The proportional hazards assumption was confirmed using time-dependent covariates or the Schoenfeld residuals plot and Schoenfeld residuals test.^20^ All independent variables met the assumption in all models. The presence of effect modification was tested by inserting a first-order interaction term into the appropriate regression models. We examined the first-order interaction in all models between the six phenotypes of the metabolic health and obesity status and life-style variables, smoking status, exercise status, or drinking status, in Table 2 and Table 3. The likelihood ratio test was used to determine the significance of interaction term in Cox proportional-hazards model. None of these interactions were statistically significant. Multicollinearity was assessed using the variance inflation factor.^21^ There was no evidence of multicollinearity. We checked outliers by plotting the likelihood displacement values and LMAX values for all independent variables.^22^ Outliers were not detected in any model. We calculated the 95% confidence interval (CI) for each hazard ratio (HR). All *P*-values were two-tailed and considered statistically significant if the values were less than 0.05. Statistical analyses were performed using IBM SPSS Statistics 22.0 (IBM Corp., Armonk, NY, USA) and Stata MP, version 14.0 (StataCorp., College Station, TX, USA).

## RESULTS

### Baseline characteristics

The baseline characteristics of the study subjects by the development of consecutive proteinuria are summarized in Table 1. Subjects who had developed consecutive proteinuria during the follow-up period tended to have higher levels of BMI, SBP, DBP, fasting plasma glucose, eGFR, and triglycerides, as well as lower levels of HDL-cholesterol. They also had a higher prevalence of hypertension, higher proportion of current smokers, and lower regular leisure-time physical activity.

**Table 1.** Baseline characteristics of study subjects according to whether consecutive proteinuria developed during the 11-year follow-up period

	Total	Developed consecutive proteinuria		*P*

		(−)	(+)	
Number	9,185	8,795	390	
Age, years	48.2 (4.2)	48.2 (4.2)	48.2 (4.1)	0.735
BMI, kg/m^2^	23.2 (2.8)	23.2 (2.8)	23.9 (3.4)	<0.001
Systolic blood pressure, mm Hg	127.6 (17.6)	127.3 (17.5)	133.4 (20.3)	<0.001
Diastolic blood pressure, mm Hg	79.7 (11.8)	79.5 (11.7)	82.5 (14.0)	<0.001
Hypertension, %^a^	28.1	27.6	40.8	<0.001
Fasting plasma glucose, mg/dL	97.2 (9.2)	97.1 (9.2)	98.5 (9.6)	0.003
Estimated glomerular filtration rate, mL/min/1.73 m^2^	84.9 (14.1)	84.7 (14.0)	87.7 (15.3)	<0.001
Triglyceride, mg/dL	111 (78–165)	110 (77–163)	137 (93–191)	<0.001
HDL-cholesterol, mg/dL	56.6 (14.9)	56.7 (14.8)	53.2 (15.2)	<0.001
Smoking habits				
Current smokers, %	57.4	56.8	71.0	<0.001
Past smokers, %	21.4	21.8	13.1	
Non-smokers, %	21.1	21.4	15.9	
Regular leisure-time physical activity, %	17.9	18.1	13.1	0.005
Average daily alcohol consumption^b^				
Non-drinkers, %	14.9	14.8	16.9	<0.001
0.1–23.0 g ethanol/day, %	42.0	42.4	34.1	
23.1–46.0 g ethanol/day, %	31.7	31.7	32.6	
≥46.1 g ethanol/day, %	11.4	11.1	16.4	

BMI, body mass index; HDL-cholesterol, high-density lipoprotein cholesterol.

Data are %, mean (standard deviation), or median (interquartile range).

^a^Hypertension was defined as systolic blood pressure ≥140 mm Hg or diastolic blood pressure ≥90 mm Hg.

^b^Average daily alcohol consumption (the number of days per week) × (the quantity of alcohol per drinking day)/7.

### Prospective analysis

#### The six phenotypes of metabolic health and obesity status and consecutive proteinuria

We analyzed the association of the six phenotypes of metabolic health and obesity status with consecutive proteinuria. During the 11-year of follow up, 390 subjects developed consecutive proteinuria, as shown in Table 2. Compared with the metabolically healthy non-obesity, metabolically healthy obesity was not associated with the risk of consecutive proteinuria (HR 0.86; 95% CI, 0.37–1.99). On the other hand, metabolic unhealthy non-obesity with ≥2 metabolic syndrome components (HR 1.77; 95% CI, 1.30–2.42), metabolic unhealthy obesity with one component (HR 1.71; 95% CI, 1.12–2.61), and metabolic unhealthy obesity with ≥2 metabolic syndrome components (HR 2.77; 95% CI, 2.01–3.82) were associated with the risk of consecutive proteinuria. The *P*-value of the interaction of the six phenotypes of metabolic health and obesity status with smoking status, exercise status, or drinking status was 0.872, 0.571, and 0.752, respectively.

**Table 2.** Multiple-adjusted HRs of the incidence of consecutive proteinuria according to the six phenotypes of the metabolic health and obesity status

	Incidence rate (per 1,000 person-years)	Cases/person-years	Crude HR (95% CI)	*P*	Multiple-adjusted HR^d^ (95% CI)	*P*
Non-obesity^a^						
Metabolic health	3.2	64/19946	1.00 (reference)		1.00 (reference)	
Metabolic unhealth with component^c^ =1	3.7	84/22850	1.14 (0.82–1.58)	0.426	1.17 (0.85–1.62)	0.342
Metabolic unhealth with components^c^ ≥2	5.8	110/18847	1.81 (1.33–2.47)	<0.001	1.77 (1.30–2.42)	<0.001
Obesity^b^						
Metabolic health	2.5	6/2417	0.77 (0.34–1.79)	0.548	0.86 (0.37–1.99)	0.729
Metabolic unhealth with component^c^ =1	5.1	33/6474	1.59 (1.04–2.42)	0.031	1.71 (1.12–2.61)	0.013
Metabolic unhealth with components^c^ ≥2	8.4	93/11126	2.60 (1.89–3.57)	<0.001	2.77 (2.01–3.82)	<0.001

BMI, body mass index; CI, confidence interval; HDL, high-density lipoprotein; HR, hazard ratio.

The interaction between the six phenotypes of the metabolic health and obesity status and smoking status, exercise status, or drinking status was 0.872, 0.571, and 0.752, respectively.

^a^Non-obesity was defined as BMI <25.0.

^b^Obesity was defined as BMI ≥25.0.

^c^Metabolic syndrome components were (1) triglyceride ≥150 mg/dL, (2) HDL-cholesterol <40 mg/dL, (3) systolic blood pressure ≥130 mm Hg or diastolic blood pressure ≥85 mm Hg, or (4) fasting plasma glucose ≥100 mg/dL.

^d^Adjusted for age, estimated glomerular filtration rate, smoking habits (non-smokers, past smokers, current smokers), regular leisure-time physical activity (yes/no), average daily alcohol consumption (non-drinkers, 0.1–23.0, 23.1–46.0, and ≥46.1 g ethanol/day).

#### The six phenotypes of metabolic health and obesity status and persistent proteinuria

We assessed the association of the six phenotypes of metabolic health and obesity status with persistent proteinuria. During the 11-year of follow up, 147 subjects developed persistent proteinuria, as shown in Table 3. These results were similar to the association of the six phenotypes of metabolic health and obesity status with consecutive proteinuria as above. Compared with the metabolically healthy non-obesity, metabolically healthy obesity was not associated with the risk of persistent proteinuria (HR 1.09; 95% CI, 0.25–4.74). On the other hand, metabolically unhealthy non-obesity with ≥2 metabolic syndrome components (HR 2.30; 95% CI, 1.30–4.07), metabolically unhealthy obesity with one component (HR 4.17; 95% CI, 2.21–7.87), and metabolically unhealthy obesity with ≥2 metabolic syndrome components (HR 4.18; 95% CI, 2.36–7.41) were associated with an increased risk of persistent proteinuria. The *P*-value of the interaction of the six phenotypes of metabolic health and obesity status with smoking status, exercise status, or drinking status was 0.618, 0.824, and 0.951, respectively.

**Table 3.** Multiple-adjusted HRs of the incidence of persistent proteinuria according to the six phenotypes of the metabolic health and obesity status

	Incidence rate (per 1,000 person-years)	Cases/person-years	Crude HR (95% CI)	*P*	Multiple-adjusted HR^d^ (95% CI)	*P*
Non-obesity^a^						
Metabolic health	0.8	17/20238	1.00 (reference)		1.00 (reference)	
Metabolic unhealth with component^c^ =1	1.1	25/23286	1.30 (0.70–2.41)	0.402	1.27 (0.69–2.36)	0.447
Metabolic unhealth with components^c^ ≥2	2.1	41/19319	2.58 (1.46–4.54)	0.001	2.30 (1.30–4.07)	0.004
Obesity^b^						
Metabolic health	0.8	2/2442	0.96 (0.22–4.14)	0.953	1.09 (0.25–4.74)	0.905
Metabolic unhealth with component^c^ =1	3.4	22/6559	4.02 (2.13–7.57)	<0.001	4.17 (2.21–7.87)	<0.001
Metabolic unhealth with components^c^ ≥2	3.5	40/11524	4.17 (2.36–7.35)	<0.001	4.18 (2.36–7.41)	<0.001

BMI, body mass index; CI, confidence interval; HDL, high-density lipoprotein; HR, hazard ratio.

The interaction between the six phenotypes of the metabolic health and obesity status and smoking status, exercise status, or drinking status was 0.618, 0.824, and 0.951, respectively.

^a^Non-obesity was defined as BMI <25.0.

^b^Obesity was defined as BMI ≥25.0.

^c^Metabolic syndrome components were (1) triglyceride ≥150 mg/dL, (2) HDL-cholesterol <40 mg/dL, (3) systolic blood pressure ≥130 mm Hg or diastolic blood pressure ≥85 mm Hg, or (4) fasting plasma glucose ≥100 mg/dL.

^d^Adjusted for age, estimated glomerular filtration rate, smoking habits (non-smokers, past smokers, current smokers), regular leisure-time physical activity (yes/no), average daily alcohol consumption (non-drinkers, 0.1–23.0, 23.1–46.0, and ≥46.1 g ethanol/day).

## DISCUSSION

In this prospective analysis, we demonstrated that, compared with metabolically healthy non-obesity, metabolically healthy obesity was not associated with an increased risk of consecutive and persistent proteinuria, but metabolic unhealth with ≥2 components was associated with an increased risk of consecutive and persistent proteinuria regardless of obesity. In this study, the definition of metabolic health had no metabolic components. Outcomes were consecutive and persistent proteinuria to exclude transient proteinuria detected by chance as much as possible. These associations were independent of age, eGFR, smoking habits, regular leisure-time physical activity, and average daily alcohol consumption at baseline.

To our knowledge, only one retrospective study reported the association between metabolically healthy obesity and the risk of proteinuria.^8^ In a retrospective study of 3,136 Japanese middle-aged men and women by the Oike Health Survey for 8 years, Hashimoto et al reported that metabolically healthy obesity was not significantly associated with the risk of proteinuria.^8^ As they defined metabolic health as the presence of one or no metabolic syndrome component, it was impossible to accurately evaluate the association between metabolically healthy obesity and the risk of proteinuria. Moreover, although their study data were based on yearly routine health examination, they used only data at baseline examination and 8-year examination. They did not use consecutive or persistent proteinuria, and they could not exclude chance proteinuria.

Obesity has been reported to be associated with the risk of proteinuria.^23^^–^^25^ In our study, metabolically healthy obesity, which was defined as no metabolic syndrome components other than waist circumference with BMI ≥25.0 kg/m^2^, was not associated with the risk of consecutive and persistent proteinuria, but metabolically unhealthy obesity, which was defined as at least one component with BMI ≥25.0 kg/m^2^, was associated with the risk of consecutive and persistent proteinuria compared with the metabolically healthy non-obesity. Therefore, our results suggested that metabolic syndrome components, excluding waist circumference, might be more important risk factors for future incident proteinuria than obesity in Japanese middle-aged men.

In our study, we did not use the composite outcome of the combination of proteinuria and reduced eGFR, because an increased eGFR, which means glomerular hyperfiltration in the early stage of renal damage, is associated with an increased risk of incident proteinuria.^26^ If we had the composite of proteinuria and reduced eGFR, incident cases would have included both subjects with eGFR<60 mL/min/1.73 m^2^ and those with eGFR ≥60 mL/min/1.73 m^2^. Therefore, we believe that use of the definition of CKD including both eGFR <60 mL/min/1.73 m^2^ and proteinuria simultaneously is inappropriate.

One of the strengths of this study is that we examined the association of the six phenotypes of metabolic health and obesity status with the risk of consecutive and persistent proteinuria during the follow-up to exclude transient proteinuria as much as possible. In our study, metabolic health was defined as no metabolic syndrome components other than waist circumference. To our knowledge, our investigation is the first prospective study to evaluate the association between metabolically healthy obesity and proteinuria.

In the present study, we did not assess the mechanism to explain the association of the six phenotypes of metabolic health and obesity status and the risk of consecutive with persistent proteinuria. The results of our study showed that metabolic unhealth with ≥2 components of metabolic syndrome, regardless of obesity, was associated with an increased risk of consecutive and persistent proteinuria. In both obese and non-obese subjects, metabolic health has been reported to result in higher insulin sensitivity and adiponectin levels than metabolic unhealth.^27^^–^^29^ Furthermore, lower insulin sensitivity and adiponectin levels were reported to be related with a higher 24-hour proteinuria level.^30^ A potential explanation that connects the association of the six phenotypes of metabolic health and obesity status with the risk of consecutive and persistent proteinuria might be in part related with insulin resistance and adiponectin, although we did not have these data. Further study will be needed to explore the six phenotypes of metabolic health and obesity status, insulin resistance, and adiponectin in association with the risk of the incident proteinuria.

This study has some limitations. First, all subjects in this study were middle-aged Japanese male workers who were registered employees of the same company and of a single ethnic group. Thus, the results of this study may not be representative of the general population in Japan, and whether we can extend these results to women, elderly men, and other ethnic groups is unclear. Second, we adjusted all models for age, eGFR, smoking habits, regular leisure-time physical activity, and average daily alcohol consumption at baseline. However, other unmeasured or unknown confounding variables, such as waist circumstance, insulin resistance, and dietary factors, may explain the associations that we observed of the six phenotypes of the metabolic health and obesity status with the risk of proteinuria. Third, proteinuria was measured using a dipstick, but dipstick tests are more likely to yield false-positive and false-negative results than specific laboratory methods. However, dipstick tests are convenient and easy to perform in clinical practice and large epidemiological studies. Fourth, inclusion of some subjects who had started to take medications, such as hypoglycemic medications, insulin, antihypertensive medications, or lipid-lowering medications, during the follow-up period may have contributed to an underestimation of the association between metabolically healthy obesity and proteinuria.

In conclusion, metabolically healthy obesity was not associated with the risk of proteinuria, but metabolic unhealthy, regardless of obesity, was associated with the risk of proteinuria. Further research investigating the mechanism of the association of the six phenotypes of metabolic health and obesity status with the risk of proteinuria is necessary.
